# Elevated Levels of sLAG-3 as a Possible Biomarker in Graves’ Disease With and Without Thyroid Eye Disease: A Prospective Observational Case–Control Study

**DOI:** 10.3390/medicina61091664

**Published:** 2025-09-13

**Authors:** Katarzyna Cieplińska, Emilia Niedziela, Edyta Jagielska, Iwona Pałyga, Anna Słuszniak, Aldona Kowalska

**Affiliations:** 1 Department of Tumor Markers, Holy Cross Cancer Center, 25-734 Kielce, Poland; 2 Collegium Medicum, Jan Kochanowski University in Kielce, 25-317 Kielce, Poland; emilia.niedziela@onkol.kielce.pl (E.N.);; 3 Department of Endocrinology, Holy Cross Cancer Center, 25-734 Kielce, Poland

**Keywords:** thyroid eye disease, Graves’ disease, soluble lymphocyte-activation gene-3, clinical activity score, soluble immune checkpoints

## Abstract

*Background and Objectives:* The pathogenesis of thyroid eye disease (TED) is driven by interactions between orbital fibroblasts and immune cells. Lymphocyte-activation gene 3 (LAG-3) is an immune checkpoint molecule with a similar structure to the T lymphocyte CD4 receptor but with higher affinity for MHC class II, and LAG-3–MHC class II interaction inhibits T lymphocyte activity. Lymphocytes shed LAG-3, generating soluble LAG-3 (sLAG-3), whose function is unclear. We investigated sLAG-3 involvement in Graves’ disease (GD) and GD-associated TED pathogenesis. *Materials and Methods:* Patients with GD-associated TED (*n* = 47) and GD without TED (*n* = 35) were enrolled alongside 37 healthy controls (HCs). Peripheral blood serum sLAG-3 levels were measured using enzyme-linked immunosorbent assays and compared across the three groups. The effect of intravenous glucocorticosteroid (IVGC) treatment (12 weeks) on sLAG-3 concentrations in patients with GD-associated TED was monitored, and associations of sLAG-3 levels with clinical characteristics were analyzed. Disease activity before and after IVGC treatment was assessed using Clinical Activity Score. *Results:* Relative to those in HCs, serum sLAG-3 levels were significantly higher in GD patients both with (*p* < 0.001) and without (*p* = 0.0129) TED. No significant difference in sLAG-3 levels was observed between the two patient groups (*p* = 1.000), and no significant change in sLAG-3 levels was detected in patients with TED after IVGC therapy (*p* = 0.0536). *Conclusions:* The higher sLAG-3 levels in patients compared to HCs suggest that sLAG-3 dysregulation may contribute to GD and GD with orbitopathy development and the pathomechanisms underlying these conditions. Metalloproteinase-mediated cleavage of LAG-3 from the lymphocyte surface enables T lymphocyte proliferation and activation, while released sLAG-3 may enhance the immune response. Further studies of sLAG-3’s mechanisms of action are needed to establish clear cut-off values and to define the diagnostic role of sLAG-3 in GD diagnosis.

## 1. Introduction

Graves’ disease (GD) is an autoimmune disorder characterized by loss of T lymphocyte tolerance to the self-antigen, thyrotropin hormone receptor (TSH-R). In GD, stimulatory autoantibodies activate TSH-R, leading to thyroid hypertrophy, as well as dysregulated thyroid hormone production and secretion [1,2,3,4,5].

Thyroid eye disease (TED), also known as Graves’ ophthalmopathy, is an extra-thyroidal manifestation that affects approximately 30% of patients with GD, although less than 10% have severe forms of TED requiring immunosuppressive treatment. Similarly to GD, TED is an autoimmune disease. The underlying pathophysiology of TED is complex, with key factors including activated CD4^+^ T cell invasion and subsequent production of cytokines that stimulate B lymphocytes to produce autoantibody binding and activation of TSH-R and insulin-like growth factor-1 receptor (IGF-1R) expressed on the cell membrane of orbital fibroblasts (OFs). This process manifests as orbital inflammation and leads to OF differentiation into myofibroblasts or adipocytes [6,7,8,9,10,11,12,13]. The interaction between OFs and immune cells is established as the primary mechanism promoting and sustaining orbital inflammation [14,15]. A positive correlation between the extent of T and B lymphocyte infiltration and the clinical activity score (CAS) of patients with TED has also been demonstrated [9]. Immune checkpoint molecules (ICs) regulate lymphocyte immune responses affecting anti-tumor immunity, defense against pathogens, and self-tolerance.

Lymphocyte-activation gene-3 (LAG-3, CD233) is referred to as a third IC, after the U.S. Food and Drug Administration approved the combination drug relatlimab/nivolumab (anti-LAG-3/anti-PD-1) for treatment of inoperable or metastatic melanoma in 2022 [16]. LAG-3 is expressed on exhausted CD4 and CD8 lymphocytes during prolonged immune responses; its protein structure resembles that of the CD4 T cell receptor, and it also binds to the canonical ligand of this receptor, major histocompatibility complex class II (MHC class II). Furthermore, MHC class II molecules have a higher affinity for LAG-3 than CD4, and the interaction between MHC class II and LAG-3 inhibits T cell activation and proliferation, contributing to maintenance of immune tolerance. Although alternative LAG-3 ligands, including galectin-3, liver and lymph node sinusoidal endothelial cell C-type lectin, fibrinogen-like protein 1, α-synuclein preformed fibrils, and (most recently) the T cell antigen receptor (TCR)-CD3 complex, have recently been reported, there are no data establishing biological roles of their interaction with LAG-3. LAG-3 expression has also been detected on regulatory lymphocytes (regulatory T (Treg) cells and Type 1 regulatory T (Tr1) cells), Natural Killer (NK) cells, Natural Killer T (NKT) cells, B cells, and plasmacytoid dendritic cells, but knowledge of its roles in these cells is limited [16,17,18,19,20].

Soluble LAG-3 (sLAG-3) is formed when lymphocytes shed the LAG-3 receptor from their surface through the activity of two TCR-induced transmembrane metalloproteases, ADAM10 and ADAM17, thereby terminating the inhibitory LAG-3 signal. A soluble isoform of the LAG-3 protein, known as LAG-3V3, which is formed by alternative RNA splicing, has also been described [17,18,19,20]. Li et al. showed that separation of LAG-3 from the cell surface increases T cell activation, proliferation, and cytokine production; although they presented preliminary data showing that sLAG-3 has no effect on antigen-driven T cell function [21], many investigators have since aimed to determine whether sLAG-3 has a specific function in immune regulation or is simply a byproduct of T cell activation. sLAG-3 has also been studied in the context of cancer, where its importance as a marker of disease stage and a predictor of treatment response has been confirmed [22,23,24,25,26,27,28].

Recently, soluble immune checkpoint molecules (sICs), including sLAG-3, have become the focus of increasing scientific interest regarding their impact on autoimmune disease pathomechanisms [29]. The aim of this study was to evaluate the role of sLAG-3 in the pathomechanism underlying TED, by comparing its levels in patients with GD, both with and without TED, as well as in healthy control subjects (HCs). We also analyzed whether sLAG-3 levels are associated with the response to intravenous glucocorticosteroid (IVGC) treatment or disease activity. Our results demonstrate that sLAG-3 levels are higher in patients with GD than those in HCs, suggesting that sLAG-3 dysregulation may contribute to GD and orbitopathy development in this disease.

## 2. Materials and Methods

### 2.1. Study Population

**Figure 1:**
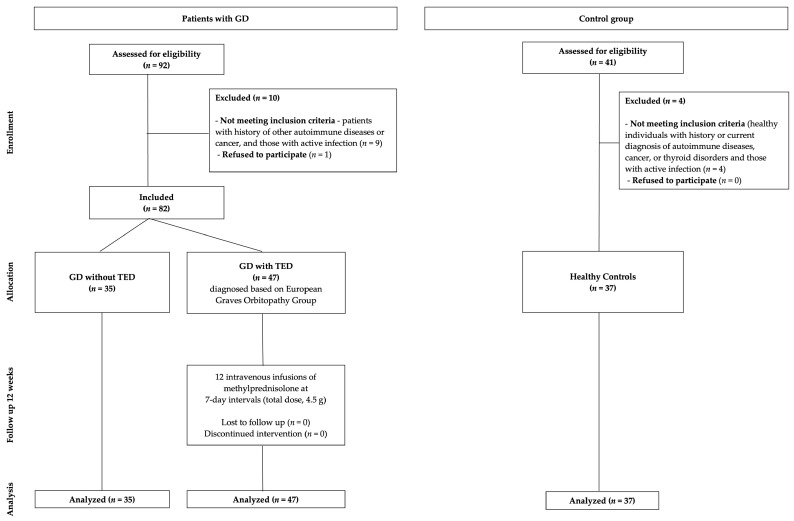
Flow diagram of participant selection. GD, Graves’ disease; TED, thyroid eye disease.

Patients with GD (*n* = 82), including 47 with TED, diagnosed based on European Graves’ Orbitopathy Group (EUGOGO) criteria, and 35 without TED, who were referred to the Department of Endocrinology at Holy Cross Cancer Centre between 1 January 2024 and 30 April 2025, were recruited to the study. Patients with a history of other autoimmune diseases or cancer, and those with active infection, were excluded from the study. Patients who had previously been treated with antithyroid drugs (ATD) were not excluded. All patients underwent a comprehensive clinical examination. TED severity was classified as mild, moderate-to-severe, or very severe based on the EUGOGO guidelines, and disease activity was assessed using CAS [7]. Data on medical history, demographic characteristics, and smoking status were collected from medical records (Table 1). According to the EUGOGO guidelines, patients with TED were qualified for treatment with IVGC and received 12 intravenous infusions of methylprednisolone at 7-day intervals (total dose, 4.5 g) [7]. To assess the effect of IVGC treatment on sLAG-3 levels, blood samples from GD patients with TED were collected before and after 12 weeks of steroid therapy. The control group included 37 healthy adults, recruited from employees of the Department of Endocrinology at the Holy Cross Cancer Center, matched for age and sex. Individuals with a history or current diagnosis of autoimmune diseases, cancer, or thyroid disorders were excluded. The study participant selection process is presented in Figure 1. All participants were informed about the purpose of the study and provided written informed consent. The study was approved by the Bioethics Committee of the Collegium Medicum of Jan Kochanowski University in Kielce (Resolution No. 18/2025) and was registered in the research registry of the Collegium Medicum of Jan Kochanowski University in Kielce.

### 2.2. Measurement and Analysis of Serum sLAG-3 Concentration

Serum sLAG-3 concentration was measured using a commercially available double-antibody sandwich enzyme-linked immunosorbent assay kit for human lymphocyte-activation gene (LAG-3) (Shanghai Sunred Biological Technology Co., Ltd., Shanghai, China) according to the manufacturer’s instructions. The material used for the analysis was serum that had been separated from whole blood by prior centrifugation and stored at −80 °C. Standards, comprising five-point serial dilutions, and samples were added to 96-well plates pre-coated with a monoclonal antibody specific for human LAG-3. Samples from HCs were analyzed simultaneously alongside patient samples. After incubation and washing, bound sLAG-3 was detected using a biotinylated anti-LAG-3 monoclonal antibody conjugated to Streptavidin-HRP, forming an immunological complex. Substrate was then added, and, after a suitable time, the reaction was stopped using stop solution. Absorbance of samples was measured at 450 nm using a microplate reader (Multiskan^®^ EX, Thermo Fisher Scientific, Waltham, MA, USA). sLAG-3 concentration (ng/mL) was calculated using a standard dilution calibration curve; detection range was 0.1–30.0 ng/mL, with a minimum detectable dose of 0.085 ng/mL.

### 2.3. Additional Tests Including Routine Thyroid Assessment

Thyroid-stimulating hormone (TSH), free thyroxine (FT4), and free triiodothyronine (FT3) were measured using the chemiluminescence method with an ARCHITECT i2000 immunoassay analyzer (Abbott Laboratories, Chicago, IL, USA). Thyroid-stimulating immunoglobulin (TSI) and interleukin 6 (IL-6) were analyzed using an IMMULITE 2000 XPi analyzer (Siemens, Erlangen, Germany). The IMMULITE 2000 TSI test is a fully automated, two-step chemiluminescent immunoassay. The IMMULITE 2000 IL-6 assay is an immunoenzymatic, sequential sandwich solid-phase test. Antithyroid peroxidase (aTPO) and anti-thyroglobulin (aTG) antibodies were measured on the COBAS e411 Analyzer (Roche Diagnostics GmbH Mannheim, Germany using electrochemiluminescence technology. Anti-TSH-R was measured on a Maglumi 1000 Analyzer (SNIBE, Shenzhen, China) using two recombinant human TSH receptors (hTSHR) in a bridging immunoassay design; the capture receptor was fixed on a solid phase (polystyrene bead), while the signal receptor, a recombinant hTSHR labeled with alkaline phosphatase, was suspended in a buffer solution. 

### 2.4. Statistical Analysis

Continuous variables are presented as means, standard deviations, medians, quartiles, and ranges (minimum and maximum), while categorical data are summarized as frequencies and percentages. The normality of distributions was verified using the Shapiro–Wilk test. The three experimental groups were compared using the chi-square test for categorical variables. Normally distributed continuous variables were analyzed using one-way ANOVA, followed by post hoc pairwise *t*-tests, with Welch’s correction applied in cases of unequal variances. The Kruskal–Wallis test was used for non-normally distributed continuous variables, with post hoc analysis performed using multiple comparisons of mean ranks for all groups. Differences in sLAG-3 concentrations before and after treatment were tested using the paired Wilcoxon signed-rank test. Due to non-normality, Spearman’s rank correlation coefficients were calculated to assess the strength of monotonic associations between sLAG-3 and CAS. Two-tailed *p* < 0.05 was considered statistically significant. A post hoc power analysis was performed for the comparison of sLAG-3 levels between GD patients and healthy controls, with effect size expressed as Cohen’s d. The diagnostic performance of sLAG-3 was evaluated by receiver operating characteristic (ROC) curve analysis with bootstrap estimation of the area under the curve (AUC) and 95% confidence intervals; the optimal cut-off point was determined by the Youden index, and sensitivity, specificity, false positives, and false negatives were reported. A sensitivity analysis including smoking status was conducted using linear regression models with sLAG-3 as the dependent variable. Finally, in the TED subgroup, a multivariable logistic regression model was fitted to assess associations between active disease (CAS ≥ 3) and sLAG-3, TSI, IL-6, age, smoking status, ATD treatment status, and GD duration. All statistical analyses were performed using Statistica software version 13.

## 3. Results

### 3.1. Patient Characteristics

Patient characteristics are summarized in Table 1. The GD with TED group comprised 30 (68.09%) women and 15 (31.92%) men, with a median (range) age of 54 (20–78) years. Mean duration of GD was 23.72 months (median (range), 12 (2–240) months), with disease duration > 12 months in 21 (44.68%) patients. TED symptoms persisted for a mean of 6.25 months (median (range), 6 (1–18) months), and the CAS was ≥3 in 41 (87.24%) patients. Of GD patients without TED, 26 (74.29%) were women and 9 (25.71%) were men, the median (range) age was 57 (20–80) years, and the mean duration of GD was 55.6 months (median 36, range (1–240) months).

### 3.2. Comparison of sLAG -3 Concentrations Among Study Groups

**Figure 2:**
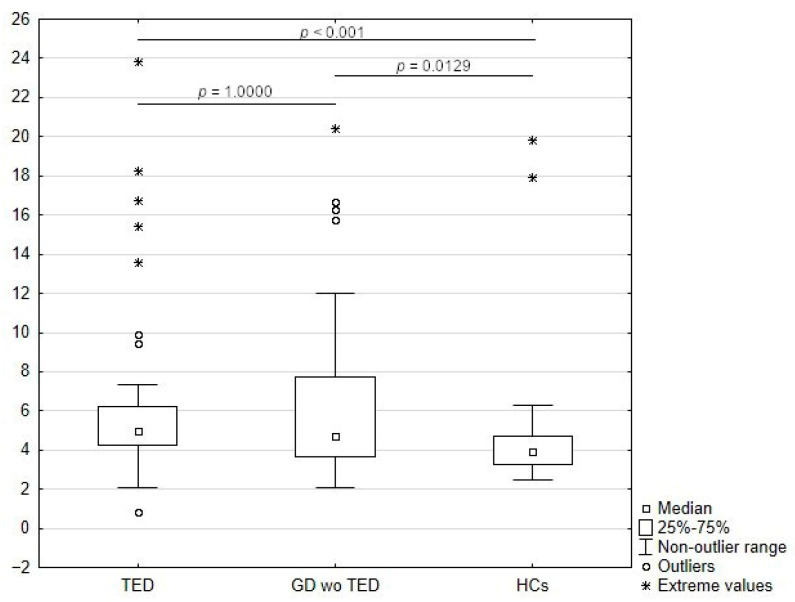
sLAG-3 levels in patients with GD with and without TED and HCs. *p*-Values were calculated using the Kruskal–Wallis test, with post hoc analysis performed using multiple comparisons of mean ranks for all groups. TED, thyroid eye disease; GD wo TED, Graves’ disease without TED; HCs, healthy controls.

Levels of sLAG-3 were significantly elevated in serum from GD patients both without (*p* = 0.0129) and with (*p* < 0.001) TED relative to those of HC; however, no significant difference between levels of sLAG-3 was detected between the two groups of patients (i.e., GD with and without TED) (*p* = 1.0000) (Table 2; Figure 2).

The post hoc power analysis comparing sLAG-3 levels between GD patients (n = 35) and healthy controls (n = 37) indicated an effect size of medium magnitude (Cohen’s d = 0.49) and a statistical power of 0.53 at α = 0.05. Thus, while differences were detectable, the study was moderately underpowered to fully exclude type II error.

**Figure 3:**
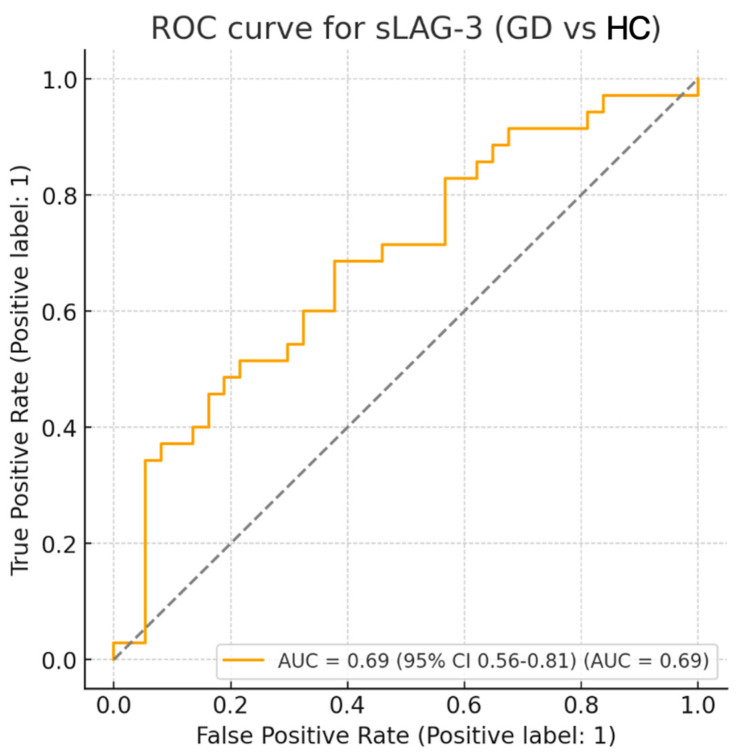
ROC curve for sLAG-3 in discriminating Graves’ disease patients (GD, *n* = 35) from healthy controls (HC, *n* = 37). The area under the curve (AUC) was 0.69 (95% CI: 0.56–0.81, *p* = 0.004). The optimal cut-off value of 4.18 ng/mL provided 68.6% sensitivity and 62.2% specificity.

ROC analysis demonstrated a moderate ability of sLAG-3 to discriminate GD patients from healthy controls (AUC = 0.69). The optimal cut-off point, determined by the Youden index, was 4.18 ng/mL, corresponding to 68.6% sensitivity and 62.2% specificity (Table 3; Figure 3).

### 3.3. Effects of IVGC Treatment

In the TED group, IVGC treatment had no significant effect on sLAG-3 levels measured after 3 months of treatment relative to those at baseline (*p* = 0.0536) (Table 4).

### 3.4. Analysis of Correlation of sLAG-3 Levels with Clinical and Laboratory Parameters

Levels of sLAG-3 did not correlate with CAS (*p* = 0.9747, rho = −0.0048), indicating that the molecule is not a useful biomarker of disease activity. Also, there was no significant correlation of sLAG-3 levels with TSI, thyroid-stimulating hormone receptor antibodies (TRAb), aTPO, aTG, TSH, FT4, or FT3 levels. Furthermore, no effects of age, duration of GD or TED, or ATD treatment on sLAG-3 levels were detected (Table 3).

In the sensitivity analysis including smoking status, no significant association was observed between smoking and sLAG-3 levels. Compared with current smokers, neither former smokers (*β* = −1.11, 95% CI: −4.41 to 2.19, *p* = 0.50) nor non-smokers (*β* = 1.99, 95% CI: −0.65 to 4.62, *p* = 0.14) showed significant differences. Adjustment for smoking did not materially change the comparison between TED and healthy controls (*β* = 0.75, 95% CI: −1.55 to 3.05, *p* = 0.52).

In the multivariable logistic regression model restricted to TED patients, none of the analyzed variables (sLAG-3, TSI, IL-6, age, smoking status, ATD status, or disease duration) showed significant association with active disease defined as CAS ≥ 3 (all *p* > 0.3) (Table 5).

## 4. Discussion

There have been few studies focused on the interaction between soluble and membrane ICs expressed on cells involved in orbital inflammation [30]. Nevertheless, previous research has demonstrated the high potential of the sPD-L1 molecule as an immunological biomarker for assessing the activity of TED, as its levels are elevated in GD patients with TED compared with GD patients without TED [31]. sPD-L1 levels correlate with CAS and are higher in patients with active TED than those with non-active TED [32,33].

Recently, there has been increasing research interest in sLAG-3, which has been extensively studied in the context of cancer, but also in conditions with autoimmune etiology. Elevated plasma concentrations of sLAG-3 are observed in patients with active immune thrombocytopenia, while its levels normalize during remission [34]. Similarly, serum sLAG-3 levels are significantly higher in patients with rheumatoid arthritis (RA); however, no significant difference in levels was detected between patients with RA in the remission phase of the disease and those with moderate to high disease activity [35]. Furthermore, sLAG-3 levels are higher in patients with antineutrophil cytoplasmic antibody-associated vasculitis and primary biliary cholangitis relative to those in healthy subjects [36,37].

Yehudai-Ofir et al. demonstrated a potential role for sLAG-3 as a predictor of acute graft-versus-host disease development [38]. In late 2024, Qinglei Yin et al. published results showing that GD patients with TED had higher levels of sLAG-3 than both GD patients without orbitopathy and HCs. Furthermore, the same study showed that the group with active TED (CAS ≥ 3) had higher sLAG-3 levels than patients with inactive TED (CAS < 2) [33]. Consistent with these results, in our cohort, we found that sLAG-3 levels were significantly elevated in both GD patients with orbitopathy (*p* < 0.001) and those without TED (*p* = 0.0129) relative to HCs; however, unlike previously published findings [33], we did not detect a significant difference in sLAG-3 levels between the two groups of patients (i.e., those with GD with or without TED (*p* = 1.0000)). In our sample, the majority of patients (87.24%) in the GD with TED group were in an active disease phase (CAS ≥ 3, median: 4); however, unlike in the Chinese study, in which patients were newly diagnosed, our patients had longer disease duration (mean: 6.25 months) and had already been treated with ATD. We hypothesize that this difference influenced the results as ATD may suppress the immune response by reducing levels of pro-inflammatory cytokines; however, the underlying mechanism remains unclear [31].

This is the first published investigation of the effect of IVGC treatment on sLAG-3 levels in TED; we noted no significant difference before and after 3 months of steroid treatment (*p* = 0.0536). Furthermore, sLAG-3 levels did not correlate with CAS, indicating that the molecule is not a useful biomarker of disease activity. Also, there was no significant correlation of sLAG-3 levels with TSI, TRAK, aTPO, or aTG levels, nor with TSH, FT4, or FT3 hormone levels. We did not observe any influence of patient age or the durations of GD or TED on sLAG-3 levels. Furthermore, cigarette smoking influences the development of thyroid disease and TED [39] but had no effect on serum sLAG-3 levels (Table 3).

Elevated sLAG-3 levels are presumably associated with increased lymphocyte activation in the GD and TED autoimmune process as T cell activation and cytokine production require LAG-3 release from the cell membrane [21]. The function of released sLAG-3 remains unclear; it may block the interaction between CD4 and TCRs with MHC class II molecules, potentially attenuating the immune response to a given antigen. Furthermore, given the high affinity of sLAG-3 for MHC class II molecules, it may compete with cellular LAG-3 to reduce its function, resulting in an enhanced lymphocyte immune response [40].

Injection of recombinant sLAG-3 into mice with gastric cancer prolonged overall survival and increased CD8^+^ T cell responses; the authors hypothesized that this was due to the increased expansion of antigen-specific CD8^+^ T cells [41]. Administration of recombinant human LAG-3 (rhLAG-3) to cultures of peripheral blood mononucleate cells collected from patients with TED led to increased secretion of pro-inflammatory cytokines (RANTES, eotaxin, and interferon-gamma-inducible protein-10) and decreased secretion of anti-inflammatory IL-10, suggesting that sLAG-3 may enhance the inflammatory feedback loop by activating antigen-presenting cells (APCs) [33]. A form of LAG-3-related therapy involves the use of LAG-3:Fc dimer (a fusion of the extracellular Ig domains of LAG-3 with the Fc section of an antibody), which stimulates dendritic cells via MHC-II molecules, thereby enhancing antigen presentation to T lymphocytes and acting as an adjuvant to APCs, potentially improving vaccine response [42,43,44].

Studies of differences in the expression of ICs on cells in orbital tissues and levels of sICs in peripheral blood will be essential to improve the understanding of the interaction of immune cells and OFs in TED pathogenesis. Recent studies show that teprotumumab, an anti-IGF-1R inhibitor, attenuates constitutive expression of MHC-II and B7 family costimulatory molecules in OFs [45].

The strengths of our study include consistent comparisons between disease groups with and without orbitopathy and the inclusion of a control group. Our study also has limitations: first, the sample size was small due to the rarity of TED; second, some of our patients had previously been treated with ATD as the majority had GD prior to TED onset. Therefore, further studies on larger samples including patients in different phases of the disease are needed. Moreover, we did not investigate the mechanisms underlying the functional impact of sLAG-3 in GD and TED. Finally, we emphasize the need to assess the phenotype of immune cells in orbital tissues in the context of ICs, together with their interaction with other cells in the orbit and sICs, to gain a better understanding of TED pathophysiology.

## 5. Conclusions

In conclusion, although the mechanism underlying the function of sLAG-3 in GD and TED remains unclear, our results demonstrate a significant increase in this molecule in patients with GD relative to that in HCs, supporting a potential role for the dysregulation of sLAG-3 in the development of GD and its extra-thyroidal manifestations, such as TED.

sICs are established to influence lymphocyte function by interacting with their ligands on the cell surface, possibly enhancing the immune response or contributing to maintenance of a state of tolerance [46]. A minimally invasive assessment of the concentrations of soluble costimulatory molecules in patient blood could provide a novel approach to diagnose autoimmune diseases, including GD and TED. Therefore, further investigation of ICs and their soluble forms is warranted.

## Figures and Tables

**Table 1:** Patient characteristics.

Characteristic	Patients with GD Without TED (N = 35)	Patients with GD and TED (N = 47)
Age (years)		
Mean (SD)	53.09 (17.78)	55.13 (12.02)
Median (Q1, Q3)	57 (39.00, 67.00)	54 (46.00, 64.00)
Range	20–80	20–78
Sex		
Female	26 (74.29%)	30 (68.09%)
Male	9 (25.71%)	15 (31.92%)
Smoking history		
Non-smoker	25 (75.76%)	20 (42.55%)
Former smoker	3 (9.10%)	11 (23.40%)
Active smoker	5 (15.15%)	16 (34.04%)
Duration of GD (months)		
Mean (SD)	55.60 (63.43)	23.72 (37.40)
Median (Q1, Q3)	36 (4.00, 72.00)	12 (7.00, 24.00)
Range	1–240	2–240
Duration of GD (months)		
≤12	12 (35.29%)	26 (55.32%)
>12	22 (64.71%)	21 (44.68%)
Duration of TED (months)		
Mean (SD)	NA	6.25 (3.98)
Median (Q1, Q3)	NA	6 (3.00, 8.00)
Range	NA	1–18
Duration of TED (months)		
≤12	NA	45 (95.75%)
>12	NA	2 (4.26%)
TSH (µIU/mL)		
Mean (SD)	1.36 (2.62)	4.49 (12.15)
Median (Q1, Q3)	0.39 (0.01, 1.41)	0.92 (0.11–1.98)
Range	0–12.49	0–71.08
TSH (µIU/mL)		
<0.35	15 (46.88%)	16 (34.04%)
0.35–4.94	14 (43.75%)	25 (53.19%)
>4.94	3 (9.38%)	6 (12.77%)
Maximum CAS at baseline		
1	NA	2 (4.25%)
2	NA	4 (8.51%)
3	NA	13 (27.66%)
4	NA	14 (29.79%)
5	NA	9 (19.15%)
6	NA	4 (8.51%)
7	NA	1 (2.13%)
Maximum CAS at baseline		
Mean (SD)	NA	3.85 (1.32)
Median (Q1, Q3)	NA	4 (3, 5)
Range	NA	1–7
TSI at baseline (IU/L)		
Mean (SD)	4.15 (4.12)	6.40 (7.93)
Median (Q1, Q3)	2.17 (1.33, 5.98)	2.90 (0.72, 10.3)
Range	0.31–15.20	0.1–37.4
TSI at baseline > 2 (IU/L)		
No	17 (48.57%)	19 (40.43%)
Yes	18 (51.43%)	28 (59.57%)
sLAG-3 at baseline (ng/mL)		
Mean (SD)	6.73 (4.52)	6.86 (5.06)
Median (Q1, Q3)	4.73 (3.63, 7.75)	4.94 (4.23, 6.23)
Range	2.10–20.43	0.848–23.84
IL-6 at baseline (pg/mL)		
Mean (SD)	3.37 (2.40)	3.30 (2.37)
Median (Q1, Q3)	2.40 (2.00, 3.36)	2.2 (2, 3.51)
Range	2–11.6	<2–12.5
IL-6 at baseline (pg/mL)		
<3.4	27 (77.14%)	29 (61.70%)
>3.4	8 (22.86%)	10 (38.30%)
Antithyroid drugs		
Treatment-naïve	2 (5.71%)	6 (12.77%)
Currently under treatment	23 (65.71%)	38 (80.85%)
Previously treated, not currently	10 (28.57%)	3 (6.38%)

GD, Graves’ disease; TED, thyroid eye disease; SD, standard deviation; Q1, first quartile; Q3, third quartile; NA, not applicable; TSH, thyroid-stimulating hormone; CAS, Clinical Activity Score; TSI, thyroid-stimulating immunoglobulin; sLAG-3, soluble lymphocyte-activation gene 3; IL-6, interleukin 6.

**Table 2:** Comparison of sLAG-3 levels in patients with GD with and without TED and HCs.

	HCs (N = 37)	Patients with GD Without TED (N = 35)	Patients with GD and TED (N = 47)	*p*-Value (HC vs. GD Without TED)	*p*-Value (HC vs. TED)	*p*-Value (GD Without TED vs. TED)
Age (years)						
Mean (SD)	55.11 (13.89)	53.09 (17.78)	55.13 (12.02)	0.5913 *	0.9945 *	0.5597 **
Median (Q1, Q3)	55 (45, 67)	57 (39, 67)	54 (46, 64)			
Range	23–78	20–80	20–78			
Sex				0.2702 ***	0.5708 ***	0.5416 ***
Female	23 (62.16%)	26 (74.29%)	32 (68.09%)			
Male	14 (37.84%)	9 (25.71%)	15 (31.92%)			
sLAG-3 at baseline (ng/mL)				0.0129 ****	<0.001 ****	1.000 ****
Mean (SD)	4.76 (3.56)	6.73 (4.52)	6.68 (5.06)			
Median (Q1, Q3)	3.94 (3.28, 4.71)	4.73 (3.63, 7.75)	5.06 (4.23, 6.16)			
Range	2.46–19.85	2.10–20.43	0.85–23.84			

* *t*-test, ** *t*-test (Welch correction), *** Chi-squared test, **** post hoc multiple comparisons of mean ranks. HCs, healthy controls; GD, Graves’ disease; TED, thyroid eye disease; SD, standard deviation; Q1, first quartile; Q3, third quartile; NA, not applicable; TSH, thyroid-stimulating hormone; CAS, Clinical Activity Score; TSI, thyroid-stimulating immunoglobulin; sLAG-3, soluble lymphocyte-activation gene-3.

**Table 3:** ROC analysis of sLAG-3 for discriminating GD patients from healthy controls.

Parameter	Value	95% CI	*p*-Value
AUC	0.69	0.56–0.81	0.004
Optimal cut-off (ng/mL)	4.18	–	–
Sensitivity (%)	68.6	–	–
Specificity (%)	62.2	–	–
False positives (n)	14	–	–
False negatives (n)	11	–	–
Youden index	0.307	–	–

**Table 4:** sLAG-3 levels in patients with TED at baseline and after 12 weeks of IVGC treatment.

At Baseline (N = 47)	sLAG-3 at Baseline (ng/mL) Mean (SD) Median (Q1, Q3) Range	sLAG-3 at 12 Weeks (ng/mL) Mean (SD) Median (Q1, Q3) Range	*p*-Value (for Difference in sLAG-3 Concentration)
All patients	6.68 (5.06)	6.91 (4.86)	0.0536
	4.94 (4.23, 6.23)	5.13 (4.62, 6.54)	
	0.85–23.84	0.79–23.84	
TSH 0.35–4. 94 (µIU/mL) (N = 25)	7.12 (5.92)	7.34 (5.61)	0.0777
	5.07 (4.19–6.44)	5.19 (4.79–6.89)	
	0.85–23.84	0.79–23.84	
TSH < 0.35 (µIU/mL) (N = 16)	6.78 (4.47)	7.05 (4.43)	0.6051
	5.19 (4.38–6.50)	5.42 (4.68–7.30)	
	2.92–18.25	3.10–17.95	
TSI > 2 (IU/L) (N = 28)	7.36 (6.07)	7.46 (5.81)	0.1213
	4.91 (4.29–5.98)	5.04 (4.61–6.64)	
	2.92–23.84	3.10–23.84	
TSI ≤ 2 (IU/L) (N = 19)	5.67 (2.89)	6.10 (2.96)	0.2432
	5.07 (4.17–6.90)	5.35 (4.68–6.54)	
	0.85–13.59	0.79–13.32	
IL-6 < 3.4 (pg/mL) (N = 29)	6.62 (4.80)	6.60 (4.52)	0.1658
	4.94 (4.23–6.09)	5.03 (4.64–5.64)	
	2.09–23.84	2.99–23.84	
IL-6 > 3.4 (pg/mL) (N = 10)	7.53 (5.98)	7.94 (5.81)	0.4413
	5.19 (4.69–7.33)	6.07 (4.68–8.69)	
	4.10–23.84	4.60–23.84	
Non-smokers (N = 20)	8.12 (6.79)	8.71 (6.64)	0.1570
	5.07 (4.29–8.61)	5.42 (4.68–9.96)	
	2.09–23.84	2.99–23.84	
Active Smokers (N = 16)	6.01 (3.43)	5.78 (2.36)	0.3794
	4.95 (4.29–5.72)	5.01 (4.61–5.99)	
	3.15–15.41	3.60–13.32	
Duration of TED ≤ 6 months (N = 28)	7.61 (6.03)	8.00 (5.95)	0.0734
	5.20 (4.21–8.38)	5.35 (4.59–9.87)	
	2.09–23.84	2.99–23.84	
Duration of TED > 6 months (N = 19)	5.30 (2.74)	5.31 (1.66)	0.3144
	4.84 (4.36–5.44)	5.13 (4.64–5.92)	
	0.85–15.41	0.79–8.83	
Duration of GD ≤ 12 months (N = 26)	6.80 (4.69)	7.29 (4.72)	0.1094
	5.14 (4.36–6.23)	5.27 (4.68–8.69)	
	2.09–23.84	2.99–23.84	
Duration of GD > 12 months (N = 21)	6.53 (5.60)	6.43 (5.11)	0.2471
	4.76 (4.10–5.44)	5.13 (4.60–5.76)	
	0.848–23.84	0.787–23.84	

sLAG-3, soluble lymphocyte-activation gene 3; SD, standard deviation; Q1, first quartile; Q3, third quartile; TSH, thyroid-stimulating hormone; TSI, thyroid-stimulating immunoglobulin; ATD, antithyroid drugs; IL-6, interleukin 6; TED, thyroid eye disease; GD, Graves’ disease; NA, not applicable; CAS, Clinical Activity Score.

**Table 5:** Multivariable logistic regression analysis of factors associated with active thyroid eye disease (CAS ≥ 3) in TED patients.

Variable	OR	95% CI	*p*-Value
lag3	0.99	0.85–1.15	0.89
TSI	1.04	0.93–1.17	0.46
IL6	1.00	0.72–1.39	1.00
Age	1.02	0.96–1.10	0.51
Smoking (former vs. current)	1.98	0.23–17.1	0.54
Smoking (never vs. current)	0.44	0.07–2.76	0.38
ATD status (former vs. current)	–	–	1.00 *
ATD status (never vs. current)	0.46	0.01–16.2	0.67
GD duration (months)	1.03	0.98–1.08	0.31

* Estimates for ATD status were unstable due to small subgroup sizes.

## Data Availability

The raw data supporting the conclusions of this article will be made available by the authors upon request.

## Abbreviations

The following abbreviations are used in this manuscript:

APC	Antigen-presenting cells
ATD	Antithyroid drugs
CAS	Clinical Activity Score
EUGOGO	European Graves’ Orbitopathy Group
GD	Graves’ disease
HCs	Healthy control subjects
hTSHR	Recombinant human TSH receptors
ICs	Immune checkpoint molecules
IVGC	Intravenous glucocorticosteroid
MHC class II	Major histocompatibility complex class II
NA	Not applicable
OF	Orbital fibroblast
Q1	First quartile
Q3	Third quartile
RA	Rheumatoid arthritis
rhLAG-3	Recombinant human LAG-3
SD	Standard deviation
sICP	Soluble immune checkpoint
sICs	Soluble immune checkpoint molecules
sLAG-3	Soluble LAG-3
TCR	T cell antigen receptor
TED	Thyroid eye disease
TSH	Thyroid-stimulating hormone
TSH-R	Thyrotropin hormone receptor
TSI	Thyroid-stimulating immunoglobulin
